# Thymic stromal lymphopoietin and alarmins as possible therapeutical targets for asthma

**DOI:** 10.1097/ACI.0000000000000793

**Published:** 2021-10-01

**Authors:** Lorenzo Salvati, Laura Maggi, Francesco Annunziato, Lorenzo Cosmi

**Affiliations:** Department of Experimental and Clinical Medicine, University of Florence, Florence, Italy

**Keywords:** alarmins, anti-thymic stromal lymphopoietin, nontype 2 asthma, severe asthma, tezepelumab, thymic stromal lymphopoietin

## Abstract

Overview of epithelial cytokines, particularly thymic stromal lymphopoietin (TSLP), released by the airway epithelium and the effects of their inhibition on the outcomes of patients with asthma.

**Recent findings:**

The epithelial cytokines are early mediators at the top of the inflammatory cascade and are attractive therapeutic targets to prevent exacerbations and improve lung function in patients with type 2 and nontype 2 asthma.

**Summary:**

Clinical trials demonstrated that tezepelumab, an anti-TSLP monoclonal antibody, is a promising alternative treatment for asthma that is effective also in nontype 2 asthma. The PATHWAY and NAVIGATOR trials have assessed its effects in improving outcomes on broad clinically diverse populations. The identification of biomarkers will help to predict potential responders and help in asthma treatment personalization.

## INTRODUCTION

Asthma is a heterogeneous respiratory disease, characterized by chronic airways inflammation, reversible airflow obstruction, and bronchial hyperresponsiveness. Two main endotypes of asthma have been defined on the basis of airway inflammation: type 2 and nontype 2 asthma [1–3]. In type 2 allergic asthma, allergen exposure induces T helper (Th) 2 cells polarization characterized by interleukin (IL)-4, IL-5 and IL-13 production. These cytokines are essential for allergen-specific immunoglobulin (Ig) E switch, eosinophils activation, recruitment and survival, mast cells degranulation, airway smooth muscle contraction and mucus hypersecretion. Allergen recognition by various pattern recognition receptors (PRRs) on epithelial cells leads to the secretion of many inflammatory molecules including the alarmins and the so called epithelial cytokines, thymic stromal lymphopoietin (TSLP), IL-25, and IL-33 [4]. These early inflammatory mediators are critical to induce the activation of both innate and adaptive immune responses [4]. Among these, IL-33 is the only protein traditionally included in the alarmin family, a group of endogenous and constitutively expressed molecules that are released immediately after degranulation, cell damage or death [5]. If we compare inflammatory pathways in asthma to a battle on the field, the epithelial cytokines could resemble trench soldiers brave enough to start the fight by moving the offensive line with effects beyond a call to arms for immune cells. Innate lymphoid cells (ILCs) are tissue-resident cells on the front line bridging innate and adaptive immunity. Targeting TSLP and alarmins, at the top of the inflammatory cascade, might be the new frontier to conquer in the battle against asthma. This review firstly describes the biology of TSLP, IL-25, and IL-33 in asthma pathogenesis and their effect on both Th2 and ILC type 2 (ILC2) responses, then it presents an overview of recent and novel therapies targeting TSLP and alarmins, particularly tezepelumab, as a promising therapeutic approach in patients with asthma. 

**Box 1:**
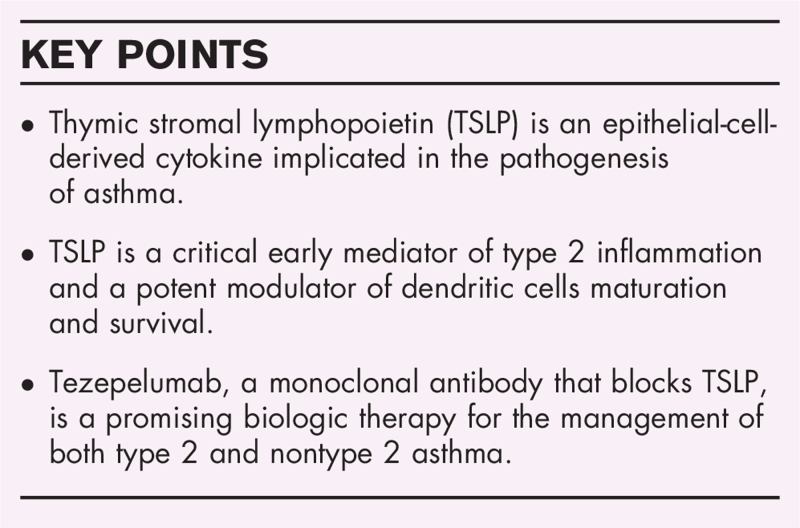
no caption available

## THYMIC STROMAL LYMPHOPOIETIN AND ALARMINS IN ASTHMA: NOT JUST A CALL TO ARMS

In response to allergens and/or to external injuries, airways epithelial cells rapidly release a wide range of molecules, including alarmins, involved in the recruitment of immune cells and in the trigger of local inflammation with the aim to activate defensive and repairing molecular mechanisms. Alarmins have an important role in inducing activation of both innate and adaptive immune responses [5].

IL-33 is one of the most representative members of the alarmin family and it is involved in asthma pathogenesis due to its key role in the activation of type 2 immune response [5]. IL-33 is localized in the nucleus of many cells (epithelial and endothelial cells, fibroblasts, adipocytes, smooth muscle cells, glial cells, hepatocytes, mast cells, myeloid cells, platelets); it bounds to a heterodimeric receptor constituted by ST-2 and IL-1RAcP and it induces a signal transduction pathway mediated by Myd88. ST-2 is expressed on a large variety of cells such as Th 2 cells, regulatory T cells (Tregs), ILC2, M2 polarized macrophages, mast cells, eosinophils, basophils, neutrophils, natural killer (NK) and invariant NK T (iNKT) cells. Most of them are critical actors for asthma development [6], due to the release of IL-4, IL-5 and IL-13 and to the induction of type 2 inflammation. Moreover, IL-33 acts on Treg cells and on ILC2 inducing the release of amphiregulin (AREG) that contributes to tissue repair [7].

IL-25 and TSLP, although not members of the alarmin family, are cytokines that act together to and share similar features with IL-33 in asthma inflammatory pathways.

IL-25, also named IL-17E, is included in the IL-17 cytokines family [8,9]. It was first identified as a type 2 cytokine produced by Th2 cells and subsequently as an epithelial cytokine. IL-25 can also be produced by ILC2, macrophages, eosinophils, basophils, as well as lung and colon epithelial cells [10]. IL-25 receptor is a heterodimer, constituted of IL17RA and IL17RB and expressed by a wide range of epithelial cells and by type 2 immune system cells; in response to IL-25, lung epithelium and fibroblast produce eotaxin, CCL17 also known as thymus and activation-regulated chemokine (TARC), and CCL22 also known as macrophage-derived chemokine (MDC). These are critical chemokines in the recruitment of eosinophils and Th2 cells [9]. In addition, IL-25 contributes to effector function of Th2 and Th9 cells and to polarization of Th2 from naïve T cells [11] and it is involved in ILC2 and iNKT cells activation [12].

TSLP is expressed by epithelial cells in the skin, airway, and ocular tissues, but also by mast cells, bronchial smooth muscle cells, dendritic cells, and fibroblasts in the airways [13,14^▪▪^,^15^]. TSLP binds to the heterodimeric receptor IL7RA and TSLPR and activates the STAT5 signalling pathway. It mediates different functions: eosinophils recruitment, basophils activation and type 2 cytokines production by Th2 cells, ILC2, and mast cells. Two variants of TSLP have been described in humans: the long isoform of TSLP (lfTSLP) promotes inflammation, whereas the short isoform (sfTSLP) has a homeostatic role [16,17].

The effects of IL-33, IL-25, and TSLP in the target cells, in most cases, are not exclusive to each single molecule, but are associated to additional stimulating conditions. For instance, the effect on Th2 cells polarization occurs in association with antigen presentation by activated dendritic cells; the effects on ILC2 is combined with IL-2 or IL-7 stimulation; the effect on mast cells activation is associated with IgE crosslinking. Moreover, it has been clearly demonstrated that IL-25, IL-33, and TSLP act synergistically on target cells [4]. In fact, they amplify type 2-driven inflammation, stimulate a positive feedback loop on cell activation due to the upregulation of their receptors, and might induce redundancy effects [4]. TSLP, IL-25, and IL-33 are cytokines associated to the airway epithelium also for their counteract effect on epithelial cells. In the airways remodelling process occurring during asthma inflammation, these cytokines could play a key role in the epithelial-mesenchimal stem cells interaction [18]. In addition, alarmins could have an effect on tight junctions regulating epithelial cell-cell interaction and epithelium integrity. In fact, IL-33 reduces the expression of claudin 1 (CLDN1) and filaggrin on keratinocytes [19,20]. IL-33 has also an indirect effect on bronchial epithelial barrier disruption mediated by IL-13 which is produced by ILC2 after IL-33 stimulation [21]. On the other hand, an opposite effect of TSLP define its ability to increase the expression of many tight junctions such as CLDN1, CLDN4, CLDN7 and occludin (OCLN), on human nasal epithelial cells [22,23]. Genetic studies have identified a large number of genes associated with asthma and its phenotypes, including IL-33 and its receptor [25–27]. All these data support the critical role of IL-25, IL-33, and TSLP in asthma pathogenesis and type 2-driven inflammation [24].

## INNATE LYMPHOID CELL TYPE 2: ON THE FRONT LINE

In the context of type 2 inflammation, Th2 cells were considered for many years the predominant effector cells triggering the pathogenic events by IL-4, IL-5 and IL-13 production. Since the recent discovery of ILC family as the innate counterpart of adaptive CD4+ T cells, the subset of ILC2 conquered a fundamental role in contributing to type 2 immune response and also in asthma pathogenesis. ILC2 are part of innate immune system, lack of antigen specific receptor, and are activated by nonspecific signals. In particular, IL-25, IL-33, and TSPL stimulate ILC2 in a synergistic manner, and they upregulate ILC2 activation which is mediated by proliferative cytokines (IL-2 and IL-7) [28]. Moreover, some TLR ligands [29] or cysteinyl leukotrienes (CysLTs), LTC4 and LTD4 activate ILC2 [30]. Following activation by different triggers, ILC2 produce type 2 cytokines and fully contribute to the physiologic immune response against helminths and parasites and to the pathogenesis of inflammatory disorders, together with Th2 cells [28,31^▪^]. Further relevant feature of ILC2 in type 2 immune response is the expression of CD40L (CD154) in response to activation mediated by IL-25 and IL-33 or TLR ligands [29]. CD40L expression by ILC2 is important to induce IgE production by B cells [29]. ILC2 are resident tissue cells and are mainly found at the mucosal barriers rather than in the peripheral blood. The location is important to profile ILC2 role in defence against pathogens, in tissue repair and in maintenance of local homeostasis. Hence it is not surprising that ILC2 are considered cells of the first defensive line for both their localization and their ‘innate’ activation modality.

## TARGETING THYMIC STROMAL LYMPHOPOIETIN IN ASTHMA: A NEW FRONTIER TO CONQUER

Despite major advances in asthma management, many patients with moderate-to-severe asthma continue to display uncontrolled disease, which means they have symptoms and exacerbations despite treatment with maximal standard-of-care therapy. Severe asthma – accounting for approximately 5–10% of all asthma cases – negatively impacts on patients’ life and contributes disproportionately to the overall burden and cost of this respiratory disease [32,33]. Available biologics are representing a game-changing in the treatment of severe asthma, however, approved monoclonal antibodies block specific type 2 pathways downstream in the inflammatory cascade without effects on the initiating events of asthma pathogenesis. Notwithstanding that there is still an unmet need for novel therapies effective in patients with nonallergic noneosinophilic severe asthma [40]. Targeting TSLP – upstream mediator triggered at the beginning of the inflammatory response – has emerged as a promising alternative treatment in patients with severe asthma [34]. Starting from the seminal work of Gauvreau *et al.* in 2014, clinical studies of tezepelumab, a first-in-class human IgG2 monoclonal antibody that binds specifically to TSLP, blocking it from interacting with its heterodimeric receptor, have shown effects in a broad population of patients with uncontrolled asthma [35–38,39^▪▪^]. In fact, blocking the various functions mediated by TSLP has been proven effective in reducing asthma exacerbations in both type 2 and nontype 2 asthma [36,39^▪▪^]. In 2017 Corren *et al.* reported on the results of PATHWAY trail (ClinicalTrials.gov number, NCT02054130) a randomized, double-blind, placebo-controlled (RDBPC) phase 2, dose-ranging, proof-of-concept study of tezepelumab [36]. Among adult patients treated with long-acting β2 agonists (LABA) and medium-to-high doses of inhaled corticosteroids (ICS), those who received subcutaneous tezepelumab had lower rates of clinically significant asthma exacerbations than those who received placebo, independent of baseline blood eosinophil counts [36]. The prebronchodilator forced expiratory volume in 1 s (pre-BD FEV1) at week 52 was higher in patients treated with tezepelumab compared to the placebo group. Remarkably, the subgroup showing the highest difference of pre-BD FEV1 change from baseline at 1 year compared to placebo was that of patients with Th2 status receiving high-dose tezepelumab, followed by patients with fraction of exhaled nitric oxide (FeNO) ≥24 ppb receiving medium-dose tezepelumab [36]. A substantial positive effect both on blood eosinophil counts and FeNO was shown, but a concern was, already at that time, the potential increased risk of infections resulting from impaired host defence secondary to inhibition of TSLP function [41]. Nasopharyngitis and bronchitis occurred in 12.9% and 5.3% of patients treated with tezepelumab, almost similar to the frequencies reported in the placebo group (11.6% and 5.1%, respectively). Pharmacokinetic (PK) and pharmacodynamic models predicted that subcutaneous tezepelumab 210 mg dose every 4 weeks associated with approximately 90% of the maximum drug effect on asthma exacerbation rate and FeNO [42]. A post hoc analysis of PATHWAY trial showed that tezepelumab reduced the rate of asthma exacerbations requiring hospitalizations or emergency department visits during the 52 weeks study time compared to placebo; treated patients spent fewer days in the hospital and in the intensive-care units [43]. In addition, tezepelumab reduced exacerbations and reduced type 2 inflammatory biomarkers in patients with and those without nasal polyposis compared to placebo [38]. Tezepelumab had a positive impact on patient reported outcomes as assessed by specific questionnaires indicating improvements in symptoms severity [44].

In 2021 Menzies-Gow *et al.* published the results of NAVIGATOR trial (ClinicalTrials.gov number, NCT03347279) a phase 3 RDBPC study aimed to further assess the potential efficacy of tezepelumab in adults and adolescents with a broad range of severe asthma phenotypes, including those with low blood eosinophil counts [39^▪▪^,^45^]. In this study, 1061 patients aged 12–80 years with severe, uncontrolled asthma underwent randomization (529 were assigned to receive tezepelumab 210 mg subcutaneously every 4 weeks and 532 to receive placebo) [39^▪▪^]. Patients who received tezepelumab had significantly fewer exacerbations and better lung function, asthma control, and health-related quality of life than those who received placebo [39^▪▪^]. The observed reductions in exacerbations occurred independently from baseline blood eosinophil count, confirming the efficacy of tezepelumab also in nontype 2 asthma [39^▪▪^]. At week 52, patients treated with tezepelumab showed significant greater clinical improvement compared to those receiving placebo as assessed by higher change in pre-BD FEV1 and improved scores on asthma control, quality of life and symptoms questionnaires [39^▪▪^]. Concomitant reductions in blood eosinophil counts and levels of FeNO in patients receiving tezepelumab occurred early (at week 2) and were sustained throughout the entire treatment period [39^▪▪^]. Serum IgE levels decreased slower during the course of treatment (at week 12) with significant decrease at week 24 compared to placebo [39^▪▪^]. The frequencies (77.1% vs 80.8%) and types of adverse events did not differ meaningfully between tezepelumab and placebo groups [39^▪▪^]. Most common adverse events were nasopharyngitis, upper respiratory tract infection, and headache [39^▪▪^]. The rate of serious infections among the two groups was comparable, particularly taking into account respiratory infections and gastrointestinal infections [39^▪▪^]. Incidence of neoplasms was similar between the two groups [39^▪▪^]. No cases of Guillain-Barré syndrome were reported [39^▪▪^]. Among patients treated with tezepeplumab none had anaphylactic reaction [39^▪▪^].

At week 52, patients entered a 12-week posttreatment follow-up period or the long-term extension study (DESTINATION; ClinicalTrials.gov number, NCT03706079) which is still underway at the time this review is written [46]. Recently completed clinical studies include the followings: the trial NCT02698501 (UPSTREAM) to determine whether anti-TSLP decreases airway hyperresponsiveness in asthmatic patients already on daily treatment with ICS; the trial NCT03406078 (SOURCE) to evaluate the oral corticosteroid-sparing potential of tezepelumab in patients with oral corticosteroid-dependent asthma; the trial NCT04048343 (NOZOMI) to assess safety of tezepelumab in Japanese adults and adolescents with inadequately controlled severe asthma [40,45,47].

A phase 2 bronchoscopy study, CASCADE (ClinicalTrials.gov number, NCT03688074), has been conducted to evaluate the effect of anti-TSLP therapy on airway inflammation and airway remodelling in patients across the spectrum of type 2 airway inflammation with fundamental results [38]. Compared to placebo group, patients treated with tezepelumab showed a significantly greater reduction of eosinophils in airway submucosal irrespective of high or low type 2 inflammation status at baseline with concomitant reductions in serum IL-5 and IL-13, and FeNO [48^▪▪^]. No significant differences between treatment groups were observed in neutrophils, CD3+ T cells, CD4+ T cells, tryptase+ mast cells, chymase+ mast cells, as well as in reticular basement membrane thickness and epithelial integrity [48^▪▪^]. However, it is interesting to note that airway hyperresponsiveness to mannitol was significantly more reduced with tezepelumab than with placebo, indicating that anti-TSLP strategy could have additional benefits in asthma beyond reducing type 2 airway inflammation [48^▪▪^]. The improvements in asthma clinical outcomes observed in previous studies, might be explained, at least partially, by reductions of eosinophilic airway inflammation, as assessed by reduced eosinophils in the airway submucosal, irrespective of baseline blood eosinophil count [48^▪▪^].

Finally, inhaled anti-TSLP has been proposed as an alternative administration route and it is currently under evaluation (ClinicalTrials.gov number, NCT04410523) [37,49]. CSJ117 is a potent neutralizing antibody fragment directed against TSLP, formulated as an engineered powder in hard capsules for delivery directly to the airways via dry powder inhaler [37,49].

Considering the impact of tezepelumab in reducing exacerbations and biomarkers of inflammation in a broad population of patients with different inflammatory endotypes, the US Food and Drug Administration granted Breakthrough Therapy designation for tezepelumab in patients with severe asthma, without an eosinophilic phenotype, who are receiving ICS/LABA with or without oral corticosteroids and additional asthma controllers [50,51].

## BLOCKING INTERLEUKIN-33 AND INTERLEUKIN-25

Biologics targeting the IL-33 pathway are in clinical evaluation in adult patients with asthma. Itepekimab and etokimab are anti-IL-33 monoclonal antibodies that seem to yield positive results, whereas astegolimab and melrilimab are anti-ST2 (IL-1RL) monoclonal antibodies targeting the IL-33 receptor [52^▪^]. Itepekimab has been evaluated alone or in combination with dupilumab in moderate-to-severe, uncontrolled asthma (ClinicalTrials.gov number, NCT03387852) [53]. Etokimab has been investigated in severe eosinophilic asthma (ClinicalTrials.gov number, NCT03469934). Astegolimab has been studied in severe, uncontrolled asthma showing reduction of annual exacerbation rates at 54 weeks even in patients with low blood eosinophils. (ClinicalTrials.gov number, NCT02918019).[54^▪^] These results suggests that inhibiting IL-33 signaling might be beneficial in a broad population including patients with non-type 2 asthma. [55] Melrilimab has completed a RDBPC phase 2a study in patients with moderately severe asthma in addition to open-label background therapy of fluticasone propionate/salmeterol 500/50 mcg twice daily; during the treatment phase, ICS/LABA was switched to ICS alone for 2 weeks and the dose reduced by approximately 50% at every 2 weeks until complete discontinuation (ClinicalTrials.gov number, NCT03207243). Another study of efficacy of melrilimab in patients affected by moderate-to-severe asthma with allergic fungal airway disease has been stopped early before meeting target enrolment due to a high screen failure rate (ClinicalTrials.gov number, NCT03393806). Considering that IL-33 is a biomarker for early diagnosis of childhood asthma, it is intriguing to speculate about the effects of targeting IL-33 axis in the paediatric population [56,57].

IL-25 emerged as a key determinant of virally induced asthma exacerbations, in fact it has been demonstrated that viral infections of the airway epithelium resulted in increased release of IL-25 in asthmatic patients [58,59]. Blocking IL-25 in experimental models of allergic asthma prevents airway hyperesponsiveness both in the sensitization phase when it reduces levels of IL-5 and IL-13, eosinophil infiltration, goblet cell hyperplasia and serum IgE secretion, and even in the challenge phase of the response [60]. In preclinical studies, ABM125, an anti-IL-25 monoclonal antibody, demonstrated potent neutralizing activity against IL-25. [reference: Bartlett N GJ, Williams T, Vincent T, et al. ABM125 Anti-IL-25 Antibody Pre-Clinical Development for Viral Asthma Exacerbations Identifies IL-25 Mediated Regulation of Type-2- and AntiViral Immunity. In: C31 Mechanistic Insights Into Lung Infection. Am Thoracic Soc 2018; 197:A7759]. However, no clinical studies of anti-IL-25 monoclonal antibodies are in progress to our knowledge.

## CONCLUSION

**FIGURE 1:**
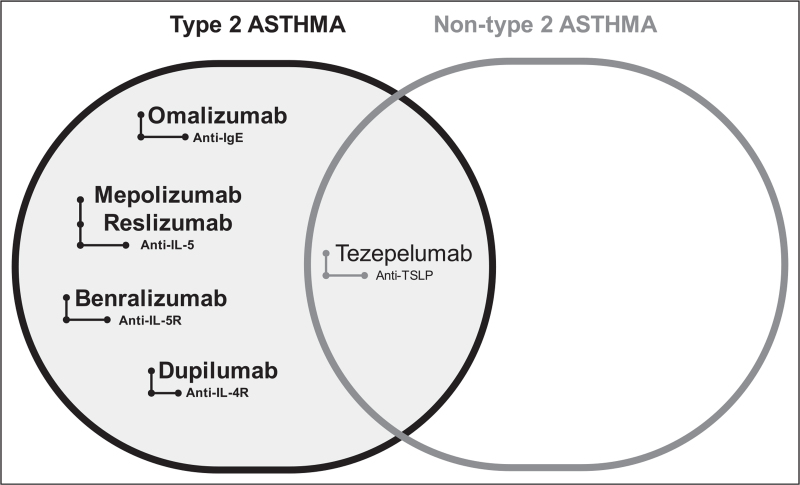
Biologics in severe asthma. Biologics (reported in bold) currently approved and used in the management of asthma have indications limited to patients with either eosinophilic or allergic phenotypes. Tezepelumab, an anti-TSLP monoclonal antibody that reduced exacerbations and improved lung function in a broad patients’ population with both type 2 and nontype 2 asthma, is a promising therapeutic approach in uncontrolled moderate-to-severe asthma irrespective of type 2 inflammatory biomarkers. TSLP, thymic stromal lymphopoietin.

Management of moderate-severe, uncontrolled asthma has taken advantage from the use of monoclonal antibodies targeting specific mediators of type 2 inflammation [61]. The role of TSLP and alarmins as early initiators of the inflammatory cascade in asthma pathogenesis has led to the development of novel targeted therapies, particularly against TSLP, with the aim of blocking the inflammatory pathway at the very beginning. More importantly tezepelumab may represent a valid therapeutic option also for nontype 2 asthma, a field in which biologics to dampen the inflammatory status are at present lacking (Fig. 1). In this regard, clinical trials of tezepelumab are showing promising results in patients with severe, uncontrolled asthma with significant reduction of exacerbations and improved outcomes. Safety of anti-TSLP must continue to be monitored in the long-term treatment both in terms of susceptibility to infections and neoplasms, even if it has been proven safe and well-tolerated in clinical studies. Research should focus on the potential modulation of the immune response secondary to TSLP inhibition. On the other hand, the identification of biomarkers for good responders will permit to select the right patient at the right time of disease for anti-TSLP therapy thus contributing to move one step forward in the battle against severe asthma.

## Acknowledgements

*None.*

### Financial support and sponsorship

*None.*

### Conflicts of interest

*There are no conflicts of interest.*

## REFERENCES AND RECOMMENDED READING

Papers of particular interest, published within the annual period of review, have been highlighted as:

▪ of special interest
▪▪ of outstanding interest

## References

[1] Gauthier M Ray A Wenzel SE. Evolving concepts of asthma. Am J Respir Crit Care Med 2015; 192:660–668. 26161792 10.1164/rccm.201504-0763PP PMC5447293

[2] Boonpiyathad T Sözener ZC Satitsuksanoa P Akdis CA. Immunologic mechanisms in asthma. Semin Immunol 2019; 46:101333. 31703832 10.1016/j.smim.2019.101333

[3] Hudey SN Ledford DK Cardet JC. Mechanisms of nontype 2 asthma. Curr Opin Immunol 2020; 66:123–128. 33160187 10.1016/j.coi.2020.10.002 PMC7852882

[4] Hong H Liao S Chen F. Role of IL-25, IL-33, and TSLP in triggering united airway diseases toward type 2 inflammation. Allergy 2020; 75:2794–2804. 32737888 10.1111/all.14526

[5] Yang D Han Z Oppenheim JJ. Alarmins and immunity. Immunol Rev 2017; 280:41–56. 29027222 10.1111/imr.12577 PMC5699517

[6] Griesenauer B Paczesny S. The ST2/IL-33 axis in immune cells during inflammatory diseases. Front Immunol 2017; 8:475. 28484466 10.3389/fimmu.2017.00475 PMC5402045

[7] Arpaia N Green JA Moltedo B. A distinct function of regulatory T cells in tissue protection. Cell 2015; 162:1078–1089. 26317471 10.1016/j.cell.2015.08.021 PMC4603556

[8] Deng C Peng N Tang Y. Roles of IL-25 in Type 2 inflammation and autoimmune pathogenesis. Front Immunol 2021; 12:691559. 34122457 10.3389/fimmu.2021.691559 PMC8194343

[9] Xu M Dong C. IL-25 in allergic inflammation. Immunol Rev 2017; 278:185–191. 28658555 10.1111/imr.12558

[10] Yao X Sun Y Wang W Sun Y. Interleukin (IL)-25: pleiotropic roles in asthma. Respirology 2016; 21:638–647. 26699081 10.1111/resp.12707

[11] Rickel EA Siegel LA Yoon BR. Identification of functional roles for both IL- 17RB and IL- 17RA in mediating IL- 25- induced activities. J Immunol 2008; 181:4299–4310. 18768888 10.4049/jimmunol.181.6.4299

[12] Stock P Lombardi V Kohlrautz V Akbari O. Induction of airway hyper-reactivity by IL- 25 is dependent on a subset of invariant NKT cells expressing IL- 17RB. J Immunol 2009; 182:5116–5512. 19342692 10.4049/jimmunol.0804213 PMC2837931

[13] Takai T. TSLP expression: cellular sources, triggers, and regulatory mechanisms. Allergol Int 2012; 61:3–17. 22270071 10.2332/allergolint.11-RAI-0395

[14▪▪] Soumelis V Liu YJ. The discovery of human TSLP as a critical epithelial cytokine in type 2 immunity and allergic disease. Nat Immunol 2020; 21:1471–1473. Erratum in: Nat Immunol. 2020 Jul 17;: PMID: 32616863. 32616863 10.1038/s41590-020-0720-7

[15] Corren J Ziegler SF. TSLP: from allergy to cancer. Nat Immunol 2019; 20:1603–1609. 31745338 10.1038/s41590-019-0524-9

[16] Dong H Hu Y Liu L. Distinct roles of short and long thymic stromal lymphopoietin isoforms in house dust mite-induced asthmatic airway epithelial barrier disruption. Sci Rep 2016; 6:39559. 27996052 10.1038/srep39559 PMC5171874

[17] Marone G Spadaro G Braile M. Tezepelumab: a novel biological therapy for the treatment of severe uncontrolled asthma. Expert Opin Investig Drugs 2019; 28:931–940. 10.1080/13543784.2019.1672657 31549891

[18] Holgate ST Holloway J Wilson S. Epithelial-mesenchymal communication in the pathogenesis of chronic asthma. Proc Am Thorac Soc 2004; 1:93–98. 16113419 10.1513/pats.2306034

[19] Ryu WI Lee H Bae HC. IL-33 down-regulates CLDN1 expression through the ERK/STAT3 pathway in keratinocytes. J Dermatol Sci 2018; 90:313–322. 29534857 10.1016/j.jdermsci.2018.02.017

[20] Seltmann J Roesner LM von Hesler FW. IL-33 impacts on the skin barrier by downregulating the expression of filaggrin. J Allergy Clin Immunol 2015; 135:1659-61.e4. 25863977 10.1016/j.jaci.2015.01.048

[21] Sugita K Steer CA Martinez-Gonzalez I. Type 2 innate lymphoid cells disrupt bronchial epithelial barrier integrity by targeting tight junctions through IL-13 in asthmatic patients. J Allergy Clin Immunol 2018; 141:300-310.e11. 28392332 10.1016/j.jaci.2017.02.038

[22] Nur Husna SM Tan HT Md Shukri N. Nasal epithelial barrier integrity and tight junctions disruption in allergic rhinitis: overview and pathogenic insights. Front Immunol 2021; 12:663626. 34093555 10.3389/fimmu.2021.663626 PMC8176953

[23] Kamekura R Kojima T Koizumi J. Thymic stromal lymphopoietin enhances tight-junction barrier function of human nasal epithelial cells. Cell Tissue Res 2009; 338:283–293. 19763625 10.1007/s00441-009-0855-1

[24] Pelaia C Crimi C Vatrella A. Molecular targets for biological therapies of severe asthma. Front Immunol 2020; 11:603312. 33329598 10.3389/fimmu.2020.603312 PMC7734054

[25] Akhabir L Sandford AJ. Genome-wide association studies for discovery of genes involved in asthma. Respirology 2011; 16:396–406. 21276132 10.1111/j.1440-1843.2011.01939.x

[26] Moffatt MF Gut IG Demenais F. GABRIEL Consortium. A large-scale, consortium-based genomewide association study of asthma. N Engl J Med 2010; 363:1211–1221. 20860503 10.1056/NEJMoa0906312 PMC4260321

[27] Han Y Jia Q Jahani PS Hurrell BP. Genome-wide analysis highlights contribution of immune system pathways to the genetic architecture of asthma. Nat Commun 2020; 11:1776. 32296059 10.1038/s41467-020-15649-3 PMC7160128

[28] Vivier E Artis D Colonna M. Innate lymphoid cells: 10 years on. Cell 2018; 174:1054–1066. 30142344 10.1016/j.cell.2018.07.017

[29] Maggi L Montaini G Mazzoni A. Human circulating group 2 innate lymphoid cells can express CD154 and promote IgE production. J Allergy Clin Immunol 2017; 139:964-976.e4. 27576126 10.1016/j.jaci.2016.06.032

[30] Qin ZL Peng YQ Fang SB. CysLT1R expression on ILC2s and effects of CysLT1R antagonist on ILC2 activity in patients with allergic rhinitis. Allergy 2020; 75:977–981. 31732973 10.1111/all.14117

[31▪] Maggi L Mazzoni A Capone M. The dual function of ILC2: From host protection to pathogenic players in type 2 asthma. Mol Aspects Med 2021; 80:100981 doi: 10.1016/j.mam.2021.100981. 34193344 10.1016/j.mam.2021.100981

[32] Backman H Jansson SA Stridsman C. Severe asthma-A population study perspective. Clin Exp Allergy 2019; 49:819–828. 30817038 10.1111/cea.13378

[33] Hekking PW Wener RR Amelink M. The prevalence of severe refractory asthma. J Allergy Clin Immunol 2015; 135:896–902. 25441637 10.1016/j.jaci.2014.08.042

[34] Porsbjerg CM Sverrild A Lloyd CM. Antialarmins in asthma: targeting the airway epithelium with next-generation biologics. Eur Respir J 2020; 56:2000260. 32586879 10.1183/13993003.00260-2020 PMC7676874

[35] Gauvreau GM O’Byrne PM Boulet LP. Effects of an anti-TSLP antibody on allergen-induced asthmatic responses. N Engl J Med 2014; 370:2102–2110. 24846652 10.1056/NEJMoa1402895

[36] Corren J Parnes JR Wang L. Tezepelumab in adults with uncontrolled asthma. N Engl J Med 2017; 377:936–946. 28877011 10.1056/NEJMoa1704064

[37] Gauvreau GM Sehmi R Ambrose CS Griffiths JM. Thymic stromal lymphopoietin: its role and potential as a therapeutic target in asthma. Expert Opin Ther Targets 2020; 24:777–792. 32567399 10.1080/14728222.2020.1783242

[38] Emson C Corren J Sałapa K. Efficacy of tezepelumab in patients with severe, uncontrolled asthma with and without nasal polyposis: a post hoc analysis of the phase 2b PATHWAY Study. J Asthma Allergy 2021; 14:91–99. 33568920 10.2147/JAA.S288260 PMC7868291

[39▪▪] Menzies-Gow A Corren J Bourdin A. Tezepelumab in adults and adolescents with severe, uncontrolled asthma. N Engl J Med 2021; 384:1800–1809. 33979488 10.1056/NEJMoa2034975

[40] Menzies-Gow A Wechsler ME Brightling CE. Unmet need in severe, uncontrolled asthma: can anti-TSLP therapy with tezepelumab provide a valuable new treatment option? Respir Res 2020; 21:268. 33059715 10.1186/s12931-020-01505-x PMC7560289

[41] Bel EH. Moving upstream – anti-TSLP in persistent uncontrolled asthma. N Engl J Med 2017; 377:989–991. 28877024 10.1056/NEJMe1709519

[42] Ly N Zheng Y Griffiths JM van der Merwe R. Pharmacokinetic and pharmacodynamic modeling of tezepelumab to guide phase 3 dose selection for patients with severe asthma. J Clin Pharmacol 2021; 61:901–912. 33368307 10.1002/jcph.1803

[43] Corren J Chen S Callan L Gil EG. The effect of tezepelumab on hospitalizations and emergency department visits in patients with severe asthma. Ann Allergy Asthma Immunol 2020; 125:211–214. 32474159 10.1016/j.anai.2020.05.020

[44] Corren J Garcia Gil E Griffiths JM. Tezepelumab improves patient-reported outcomes in patients with severe, uncontrolled asthma in PATHWAY. Ann Allergy Asthma Immunol 2021; 126:187–193. 33169672 10.1016/j.anai.2020.10.008

[45] Menzies-Gow A Colice G Griffiths JM. NAVIGATOR: a phase 3 multicentre, randomized, double-blind, placebo-controlled, parallel-group trial to evaluate the efficacy and safety of tezepelumab in adults and adolescents with severe, uncontrolled asthma. Respir Res 2020; 21:266. 33050934 10.1186/s12931-020-01526-6 PMC7550847

[46] Menzies-Gow A Ponnarambil S Downie J. DESTINATION: a phase 3, multicentre, randomized, double-blind, placebo-controlled, parallel-group trial to evaluate the long-term safety and tolerability of tezepelumab in adults and adolescents with severe, uncontrolled asthma. Respir Res 2020; 21:279. 33087119 10.1186/s12931-020-01541-7 PMC7576983

[47] Wechsler ME Colice G Griffiths JM. SOURCE: a phase 3, multicentre, randomized, double-blind, placebo-controlled, parallel group trial to evaluate the efficacy and safety of tezepelumab in reducing oral corticosteroid use in adults with oral corticosteroid dependent asthma. Respir Res 2020; 21:264. 33050928 10.1186/s12931-020-01503-z PMC7550846

[48▪▪] Diver S Khalfaoui L Emson C. Effect of tezepelumab on airway inflammatory cells, remodelling, and hyperresponsiveness in patients with moderate-to-severe uncontrolled asthma (CASCADE): a double-blind, randomised, placebo-controlled, phase 2 trial. Lancet Respir Med 2021; 9: S2213-2600(21)00226-5. doi: 10.1016/S2213-2600(21)00226-5. Epub ahead of print. 10.1016/S2213-2600(21)00226-5 34256031

[49] Gauvreau GM Hohlfeld JM Grant S. Efficacy and safety of an inhaled anti-TSLP antibody fragment in adults with mild atopic asthma. Am J Respir Crit Care Med 2020; 201:A4207.

[50] Kaplon H Reichert JM. Antibodies to watch in. MAbs 2021; 13:1860476. 33459118 10.1080/19420862.2020.1860476 PMC7833761

[51] Mullard A. Tezepelumab prepares to enter the asthma antibody fray. Nat Rev Drug Discov 2021; 20:91. 10.1038/d41573-021-00011-z 33441998

[52▪] Saikumar Jayalatha AK Hesse L Ketelaar ME. The central role of IL-33/IL-1RL1 pathway in asthma: from pathogenesis to intervention. Pharmacol Ther 2021; 225:107847. 33819560 10.1016/j.pharmthera.2021.107847

[53] Wechsler M Ruddy MK Pavord ID. SAR440340, An Anti-IL-33 Monoclonal Antibody, Demonstrated a Significant Reduction of LOAC Events and Improved Pre-BD FEV1 in Patients with Moderate to Severe Asthma: Results from the Phase 2 Proof of Concept Study. In: B101 New biological treatments for asthma. Am J Respir Crit Care Med 2020; 201: A4269.

[54▪] Kelsen SG Agache IO Soong W. Astegolimab (anti-ST2) efficacy and safety in adults with severe asthma: A randomized clinical trial. J Allergy Clin Immunol 2021; 148:790–798. 33872652 10.1016/j.jaci.2021.03.044

[55] Tamari M Trier AM Kim BS. Emerging targeted therapeutics underscore immunologic heterogeneity of asthma. J Allergy Clin Immunol 2021; 148:719–721. 34310926 10.1016/j.jaci.2021.07.008

[56] Licari A Castagnoli R Brambilla I. Asthma endotyping and biomarkers in childhood asthma. Pediatr Allergy Immunol Pulmonol 2018; 31:44–55. 30069422 10.1089/ped.2018.0886 PMC6069590

[57] Wang Y Wang L Hua S. Interleukin-33 in children with asthma: a systematic review and meta-analysis. Allergol Immunopathol 2017; 45:387–392. 10.1016/j.aller.2016.12.007 28410870

[58] Andreakos E Papadopoulos NG. IL-25: the missing link between allergy, viral infection, and asthma? Sci Transl Med 2014; 6:256fs38. 10.1126/scitranslmed.3010273 25273094

[59] Beale J Jayaraman A Jackson DJ. Rhinovirus-induced IL-25 in asthma exacerbation drives type 2 immunity and allergic pulmonary inflammation. Sci Transl Med 2014; 6:256ra134. 10.1126/scitranslmed.3009124 PMC4246061 25273095

[60] Ballantyne SJ Barlow JL Jolin HE. Blocking IL-25 prevents airway hyperresponsiveness in allergic asthma. J Allergy Clin Immunol 2007; 120:1324–1331. 17889290 10.1016/j.jaci.2007.07.051

[61] Kaur R Chupp G. Phenotypes and endotypes of adult asthma: moving toward precision medicine. J Allergy Clin Immunol 2019; 144:1–12. 31277742 10.1016/j.jaci.2019.05.031

